# Effects of ultrasound-guided alveolar recruitment manoeuvres compared with sustained inflation or no recruitment manoeuvres on atelectasis in laparoscopic gynaecological surgery as assessed by ultrasonography: a randomized clinical trial

**DOI:** 10.1186/s12871-022-01798-z

**Published:** 2022-08-16

**Authors:** Xiong-zhi Wu, Hai-mei Xia, Ping Zhang, Lei Li, Qiao-hao Hu, Su-ping Guo, Tian-yuan Li

**Affiliations:** 1 Department of Anesthesiology, First Affiliated Hospital of Nanchang University, NO. 17, Yongwaizheng Street, Nanchang, Jiangxi 330006 China; 2 Department of Anesthesiology, Shaoxing People’s Hospital (Shaoxing Hospital, Zhejiang University School of Medicine), NO.568, North Zhongxing Road, Shaoxing, Zhejiang China; 3 Department of Anesthesiology, Jiangxi Provincial People’s Hospital, NO.152 Aiguo Road, Nanchang, Jiangxi 330006 China; 4 Department of Ultrasound Medicine, First Affiliated Hospital of Nanchang University, NO. 17, Yongwaizheng Street, Nanchang, Jiangxi 330006 China

**Keywords:** General anesthesia, Ultrasound-guided, Sustained inflation, Recruitment manoeuvres, Atelectasis

## Abstract

**Background:**

The majority of patients may experience atelectasis under general anesthesia, and the Trendelenburg position and pneumoperitoneum can aggravate atelectasis during laparoscopic surgery, which promotes postoperative pulmonary complications. Lung recruitment manoeuvres have been proven to reduce perioperative atelectasis, but it remains controversial which method is optimal. Ultrasonic imaging can be conducive to confirming the effect of lung recruitment manoeuvres. The purpose of our study was to assess the effects of ultrasound-guided alveolar recruitment manoeuvres by ultrasonography on reducing perioperative atelectasis and to check whether the effects of recruitment manoeuvres under ultrasound guidance (visual and semiquantitative) on atelectasis are superior to sustained inflation recruitment manoeuvres (classical and widely used) in laparoscopic gynaecological surgery.

**Methods:**

In this randomized, controlled, double-blinded study, women undergoing laparoscopic gynecological surgery were enrolled. Patients were randomly assigned to receive either lung ultrasound-guided alveolar recruitment manoeuvres (UD group), sustained inflation alveolar recruitment manoeuvres (SI group), or no RMs (C group) using a computer-generated table of random numbers. Lung ultrasonography was performed at four predefined time points. The primary outcome was the difference in lung ultrasound score (LUS) among groups at the end of surgery.

**Results:**

Lung ultrasound scores in the UD group were significantly lower than those in both the SI group and the C group immediately after the end of surgery (7.67 ± 1.15 versus 9.70 ± 102, difference, -2.03 [95% confidence interval, -2.77 to -1.29], *P* < 0.001; 7.67 ± 1.15 versus 11.73 ± 1.96, difference, -4.07 [95% confidence interval, -4.81 to -3.33], *P* < 0.001;, respectively). The intergroup differences were sustained until 30 min after tracheal extubation (9.33 ± 0.96 versus 11.13 ± 0.97, difference, -1.80 [95% confidence interval, -2.42 to -1.18], *P* < 0.001; 9.33 ± 0.96 versus 10.77 ± 1.57, difference, -1.43 [95% confidence interval, -2.05 to -0.82], *P* < 0.001;, respectively). The SI group had a significantly lower LUS than the C group at the end of surgery (9.70 ± 1.02 versus 11.73 ± 1.96, difference, -2.03 [95% confidence interval, -2.77 to -1.29] *P* < 0.001), but the benefit did not persist 30 min after tracheal extubation.

**Conclusions:**

During general anesthesia, ultrasound-guided recruitment manoeuvres can reduce perioperative aeration loss and improve oxygenation. Furthermore, these effects of ultrasound-guided recruitment manoeuvres on atelectasis are superior to sustained inflation recruitment manoeuvres.

**Trial registration:**

Chictr.org.cn, ChiCTR2100042731, Registered 27 January 2021, www.chictr.org.cn.

## Introduction

Approximately 90% of patients develop atelectasis during general anesthesia [1]. Atelectasis is considered to be a critical factor that contributes to the development of most postoperative pulmonary complications (PPCs) [2, 3], which result in significantly higher morbidity, mortality, and increased use of hospital resources [4–7]. Especially, with the continuous development of minimally invasive surgery, laparoscopic surgery is increasingly carried out in hospitals, and pneumoperitoneum increasing the abdominal pressure and Trendelenburg position have been demonstrated to damage respiratory function during surgery, mainly worsening perioperative atelectasis [8–10]. Therefore, preventing perioperative atelectasis is a great challenge for anesthesiologists.

In many studies, lung recruitment manoeuvres (RMs) have been demonstrated to be effective in reducing perioperative atelectasis and improving oxygenation in both children and adults during laparoscopic surgery [8, 9, 11, 12]. Lung RMs are aimed at (re)open collapsed lung units and increasing end-expiratory lung volume by dynamically and transiently increasing transpulmonary pressure [13]. There are many methods of recruitment manoeuvres, including an incremental PEEP, sigh, pressure control method, sustained inflation, and so on, but it remains controversial which method is optimal [14]. The pressure threshold that can (re)open collapsed lung parts during RMs is multifactorial and cannot be calculated precisely [15, 16]. In addition, an increase in airway pressure will lead to an increase in the pressure of lung units, as well as those "open" lung units, which may be harmed by overdistention [17]. Thus, the value of RMs is controversial without any image monitoring. Some clinical studies have failed to show benefits to the results and even have had adverse side effects [18]. The benefits of RMs require a balance between reaeration and overaeration.

Lung ultrasonography is an easy-to-use, portable, noninvasive, visual, and nonradiative technique that has been widely used in clinical monitoring and diagnosis [19, 20]. Many studies [21, 22] have demonstrated that pulmonary ultrasonography can evaluate the degree of aeration loss and diagnose atelectasis accurately by using a validated semiquantitative score [21] in the perioperative period, and lung ultrasonic imaging can be conducive to confirming the effects of lung recruitment manoeuvres.

Therefore, we designed a randomized, double-blinded trial to evaluate the effects of ultrasound-guided RMs on reducing perioperative atelectasis in patients undergoing laparoscopic gynecological surgery by ultrasonography and to check whether the effects of RMs under ultrasound guidance (late-model) on atelectasis are superior to sustained inflation RMs that are classical and widely used in an adult surgical population.

## Methods

### Study design

**Fig. 1:**
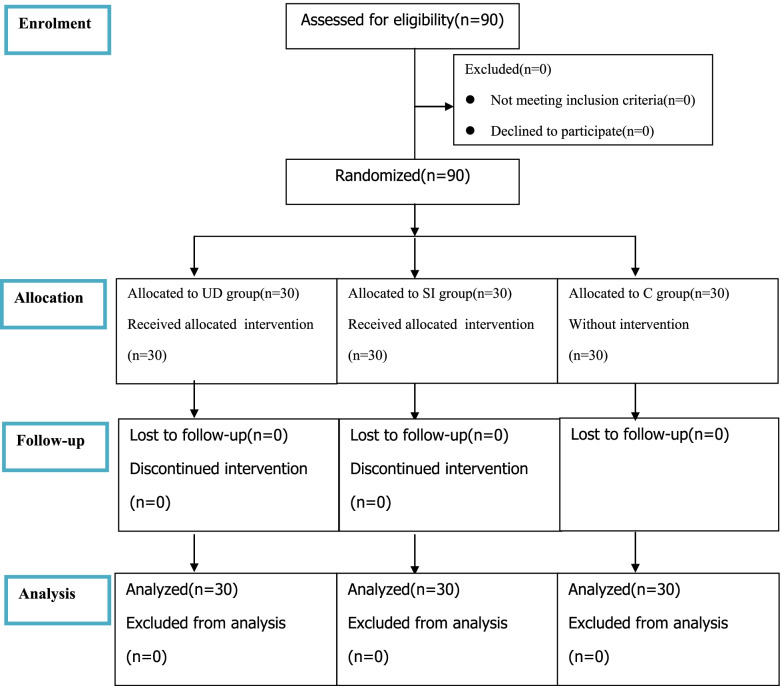
CONSORT flow diagram

This study was approved by the ethics committee of the First Affiliated Hospital of Nanchang University (NO. 2021–8-002), and written informed consent was obtained from all subjects participating in the trial. The trial was registered before patient enrollment at chictr.org.cn (trial number: ChiCTR2100042731; Principal investigator: X.Z.; Date of registration: 27/Jan/2021). The study was conducted between April 2021 and September 2021 at the First Affiliated Hospital of Nanchang University, China. The enrollment and allocation of participants are compiled in Fig. 1.

### Inclusion and exclusion criteria

The inclusion criteria were women more than 18 years old (ASA I or II) undergoing an elective laparoscopic gynecological surgery that was expected to be no more than 2 h. The following exclusion criteria were applied: patient refusal, body mass index > 35 kg/m^2^, ASA > II, previous abdominal and/or chest surgery, emergency surgery, and abnormal pulmonary function.

### Randomization and masking

Before the trial, patients were randomly assigned to receive either lung ultrasound-guided alveolar recruitment manoeuvres (UD group), sustained inflation alveolar recruitment manoeuvres (SI group), or no RMs (C group) using a computer-generated table of random numbers. Allocation details were sealed in numbered envelopes. Patients, PACU (postanesthesia care unit) teams, ultrasound assessors, and investigators who scored the LUS and statistical analysis were blinded to group allocation until the final statistical analysis was completed.

### Anesthesia protocol

No premedication was given before anesthesia. Patients were subjected to standard monitoring. After preoxygenation with 100% oxygen for 3 min, anesthesia induction was performed with standard doses of propofol, cisatracurium and sufentanil. Anesthesia was maintained by target-controlled infusion of propofol and remifentanil and repetitive doses of cisatracurium. After endobronchial intubation with a 7.0-mm cuffed tube, all individuals underwent volume-controlled ventilation with a Fabius GS premium (Drager, Germany) and received a respiratory rate of 12 breaths/min, tidal volume of 8 ml/kg of predicted body weight (PBW), positive end-expiratory pressure of 5 cmH2O, and a fraction of inspired oxygen of 0.6. Each patient underwent radial artery cannulation to monitor invasive blood pressure after induction. After ending the surgical procedure and before reversing the muscle relaxant, all individuals received bilateral transversus abdominis plane block under ultrasound guidance with each block 20 mL of ropivacaine 0.375%. To ensure the analgesic effect of these patients, postoperative pain was evaluated using a visual analog score from 0 (no pain at all) to 10 (most severe pain) at 30 min after tracheal extubation. Each individual received intravenous neostigmine 0.05 mg/kg and atropine 0.02 mg/kg to reverse the neuromuscular block before emergence. All subjects were transferred to the PACU after extubation of the endotracheal tube in the operating room. Each individual received a chest radiograph at PACU and 48 h postoperative period. During laparoscopic surgery, the patient was placed in the Trendelenburg position and pneumoperitoneum (pressure < 12 mmHg).

### Lung ultrasonography

All ultrasound examinations were performed by two trained echographic instruments (Xiongzhi-Wu and Shuping-Guo, respectively, with 1 year and 4 years of experience in lung ultrasound examinations), respectively, using a wisonic ultrasound machine (Wisonic Medical Co., Ltd., Shenzhen, China) with a 4 to 12 MHz linear transducer. Twelve quadrants (six quadrants on the left and right lungs) were assessed by ultrasonography, as described previously [21, 22]. Each hemithorax was divided into anterior, lateral, and posterior areas that were separated by the anterior and posterior axillary lines and divided into upper and lower portions that were separated by a boundary of 1 cm above the nipple. Intercostal spaces of each quadrant were scanned, and ultrasound images of this quadrant were saved for analysis. We evaluated lung aeration by using the LUS that was previously described [21] based on the following scoring criteria: ① Normal aeration (N): lung sliding sign and A-lines or less than 3 isolated B line(s), marked as N; ② Moderate aeration loss (B1): multiple, vertical, laser-like B-lines or one or more small subpleural consolidations, marked as B1; ③ Severe aeration loss (B2): multiple merged B lines occupying the whole lung image (so-called “white lung”) or multiple small subpleural consolidations, marked as B2; ④ Complete aeration loss (C): localized consolidation (subpleural tissue-like pattern), marked as C. An LUS per quadrant was assigned as follows: *N* = 0, B1 = 1, B2 = 2, and C = 3. The scores of 12 quadrants were added to calculate the total LUSs. The higher the scores are, the more serious the aeration loss.

### Study protocol

**Fig. 2:**
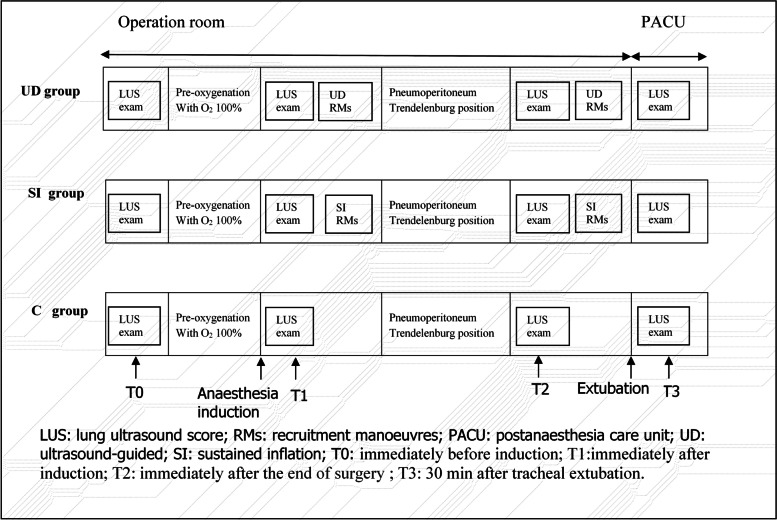
Schematic diagram of the study protocol. LUS: lung ultrasound score; RMs: recruitment manoeuvres; PACU: postanaesthesia care unit; UD: ultrasound-guided; SI: sustained inflation; T0: immediately before induction; T1:immediately after induction; T2: immediately after the end of surgery; T3: 30 min after tracheal extubation

Details of the study protocol are provided in Fig. 2. In the three groups, lung ultrasonic examination was carried out at the following four time points: immediately before induction (T0), immediately after induction (T1), immediately after the end of surgery (T2), and 30 min after tracheal extubation (T3). In both the UD group and the SI group, RMs were carried out at T1 and T2 after each lung ultrasonography. In the UD group, RMs were performed under direct real-time ultrasound guidance. A stepwise increase in airway pressure from 10 cmH_2_O by 5 cmH_2_O increments with FiO_2_ of 0.4 was applied manually until noncollapsed regions were visible on the ultrasonic imaging. The maximum airway pressure was no more than 40 cmH_2_O, and the tidal volume of each recruitment manoeuvre was limited to 20 ml/kg. In the SI group, RMs were carried out by manual inflation with 30 cmH_2_O airway pressure for 15 s. The C group had no recruitment manoeuvres.

### Primary and secondary study outcomes

The primary outcome was LUS at T2. Secondary outcomes included (1) LUS at other time points; (2) mechanical ventilation parameters: tidal volume, Ppeak, Pplateau, the modified driving pressure was calculated as Ppeak minus PEEP, SpO_2_, peripheral oxygen saturation; (3) hemodynamic parameters: pulse, mean arterial pressure (MAP), and arterial-blood-gas analysis results at T1, T2 and T3; (4) postoperative complications: atelectasis confirmed by chest radiograph at PACU and 48 h postoperative period, pneumonia, pleural effusion; (5) the number of RMs; (6) postoperative pain at the time point T3 and length of hospital stay after surgery; and (7) baseline characteristics: age, height, weight, crystalloid fluids, urine output, and blood loss.

### Rescue strategy of complications of RMs

Complications of RMs were defined as hypotension (MAP < 60 mmHg), hypertension (systolic blood pressure > 150 mmHg), SpO_2_ < 90%, and pneumothorax. If the patient has hypertension or hypotension, we could give a fluid bolus or vasoactive medication. Pneumothorax may need to require a chest tube. When SpO_2_ decreased to 90%, we needed to increase FIO_2_ to 1.0. If the above measures are invalid, the strategy can be modified according to the judgment of the attending anesthesiologist or stopped.

### Sample-size calculation

The primary analysis was to determine the difference in the mean LUS between groups. Based on a previous trial conducted in patients undergoing laparoscopic surgery [21, 22], it was estimated that 90 patients (30 per group, allowing for 15% attrition) would be required to detect a 4-point mean LUS difference among groups with 80% power and an alpha of 0.05 (two-sided). This calculation was based on an assumption of a common SD of 4.1 [21] using an analysis of variance (ANOVA).

### Statistical analysis

Statistical analyses were performed with SPSS, version 23 (SPSS Inc.). Continuous data were tested for normality using Q-Q plots. Data showing a normal distribution are presented as the mean ± SD, and data showing a nonnormal distribution are presented as the median [IQR]. Continuous variables were analysed using ANOVA or Kruskal‒Wallis tests. Categorical data are expressed as frequencies or percentages and were compared with the chi-square test or Fisher’s exact test where appropriate. All data, including the LUS and gravity-dependent changes in lung aeration per quadrant, were analysed both within and between groups by two-way repeated-measures ANOVA with Bonferroni correction. A two-sided *P* value < 0.05 was considered statistically significant.

## Results

### Baseline characteristics

There were no significant differences in baseline characteristics, including age, height, weight, PBW, duration of surgery, duration of anesthesia, urine output, and blood loss among groups. (*P* > 0.05) (Table 1). In the UD group, two patients developed hypotension during lung RMs and recovered after stopping the RMs without vasoactive medication.

**Table 1:** Comparisons of baseline characteristics in three groups

Baseline characteristics	C group	SI group	UD group	F	*P* value
Age(yrs)	50.20 ± 8.58	50.27 ± 9.03	47.20 ± 9.27	1.146	0.323
Height(cm)	158.37 ± 5.68	157.50 ± 3.82	157.30 ± 4.17	0.45	0.639
Weight(kg)	57.60 ± 7.45	55.70 ± 6.65	53.87 ± 7.11	2.085	0.131
PBW(kg)	50.93 ± 5.17	50.14 ± 3.48	49.96 ± 3.79	0.45	0.639
Duration of surgery(min)	137.33 ± 17.80	135.73 ± 17.93	137.67 ± 20.00	0.093	0.912
Duration of Anesthesia(min)	156.20 ± 17.55	153.83 ± 18.89	160.10 ± 20.09	0.844	0.434
Urine output(ml)	325.67 ± 93.76	308.67 ± 81.86	312.33 ± 128.32	0.225	0.799
Fluid infusion Volume(ml)	1363.33 ± 239.95	1490.00 ± 259.11	1446.67 ± 214.53	2.185	0.119
Blood loss(ml)	184.33 ± 48.40	202.67 ± 55.77	186.67 ± 73.03	0.831	0.439

### Primary outcome: serial LUSs

**Fig. 3:**
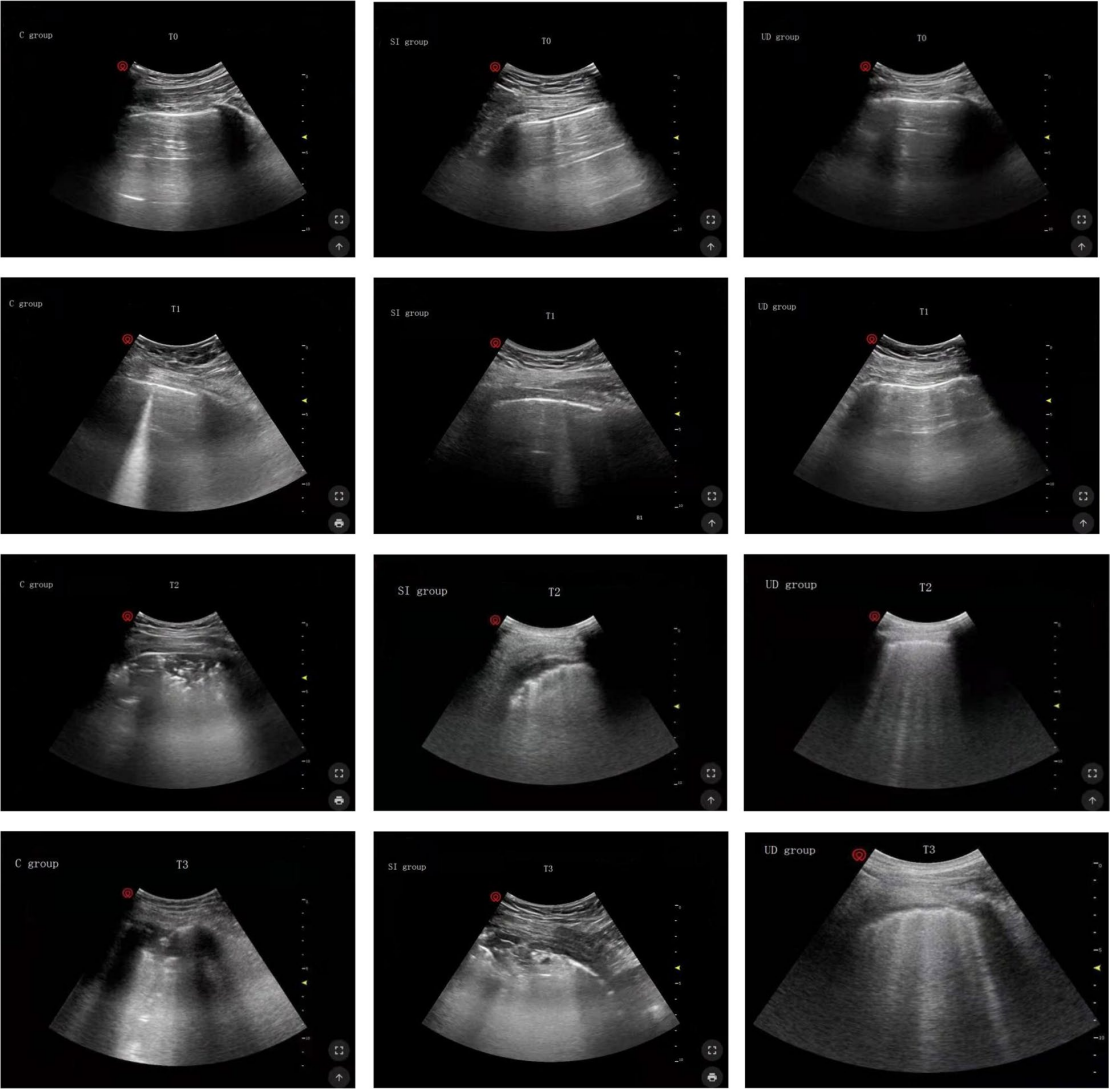
Lung ultrasound images of one representative patient in the lateral chest wall at different time points. T0: immediately before induction; T1: immediately after induction; T2: immediately after the end of surgery; T3: 30 min after tracheal extubation; C group: control group; SI group: sustained inflation recruitment manoeuvres group; UD group: ultrasound-guided recruitment manoeuvres group

**Fig. 4:**
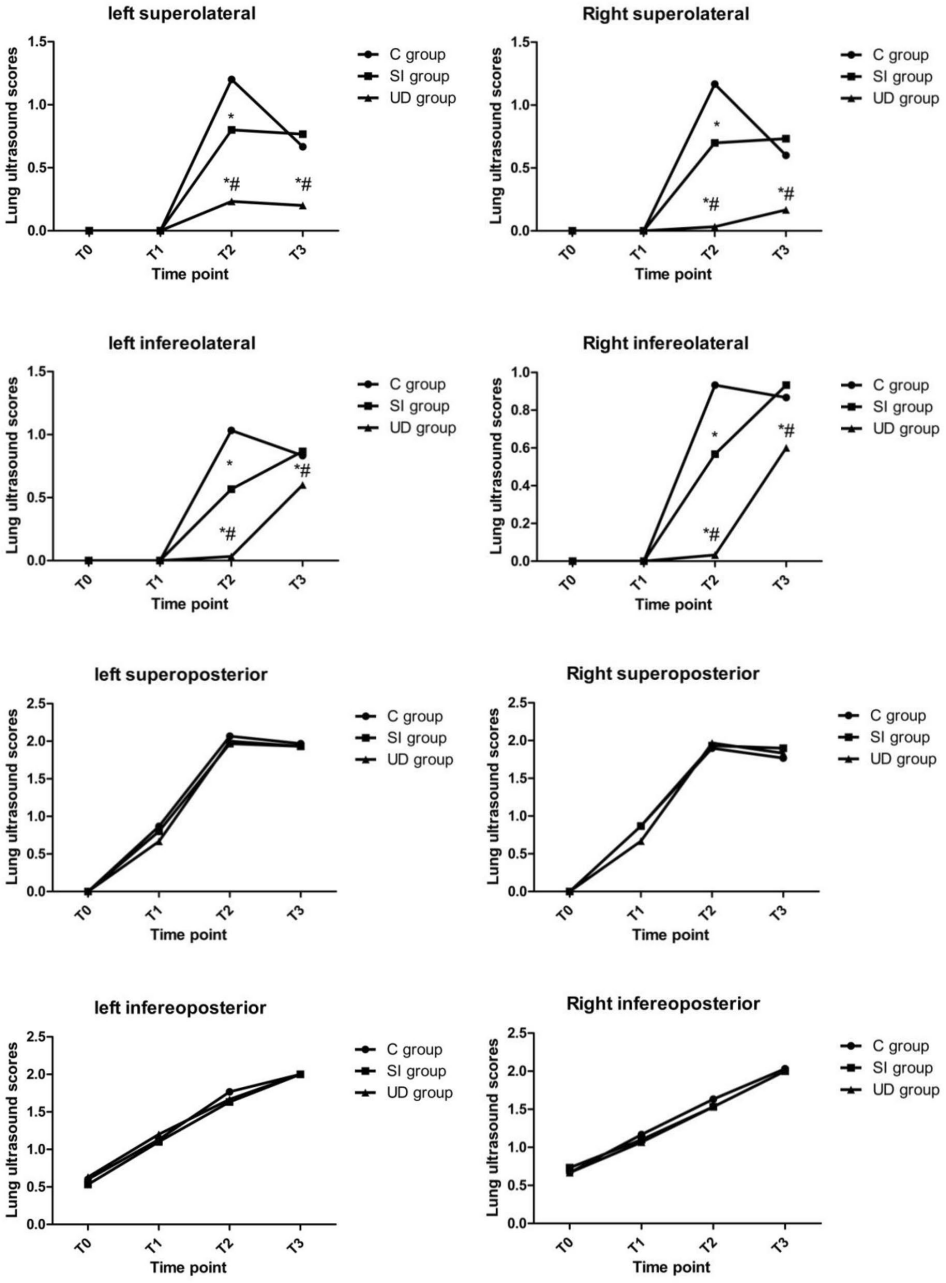
Temporal evolution of the mean LUS results per quadrant per group**.** LUS of the lung in the anterior chest area were all 0, thus no statistical analysis was made. T0: immediately before induction; T1: immediately after induction; T2: immediately after the end of surgery; T3: 30 min after tracheal extubation; C group: control group; SI group: sustained inflation recruitment manoeuvres group; UD group: ultrasound-guided recruitment manoeuvres group. *Denotes significant difference compared with C group (P < 0.05); #denotes significant difference compared with SI group (*P* < 0.05)

**Fig. 5:**
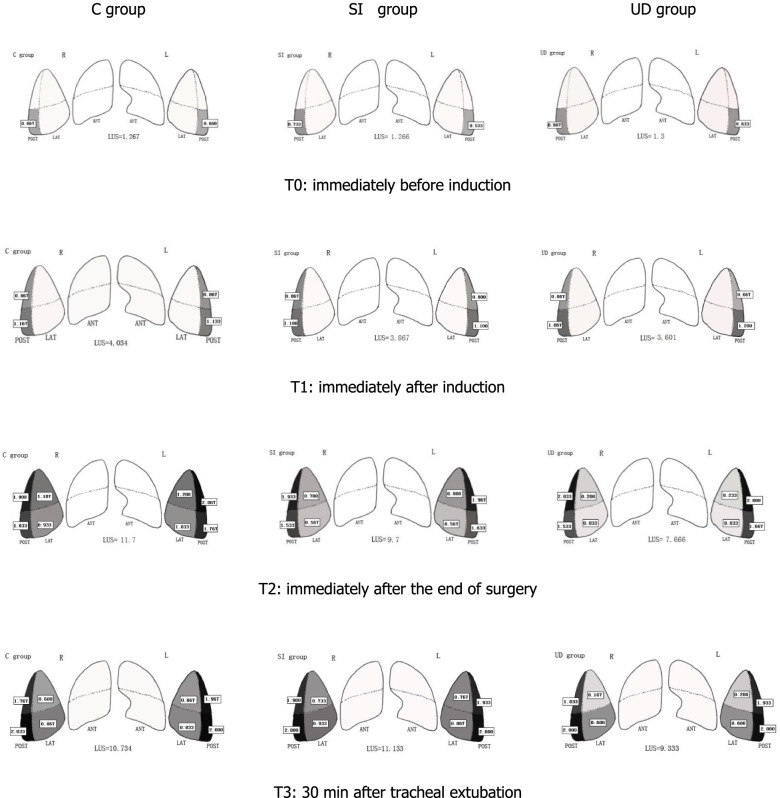
Temporal evolution of the Visual model of LUS results per quadrant per group. C group: control group; SI group: sustained inflation recruitment manoeuvres group; UD group: ultrasound-guided recruitment manoeuvres group. ANT:anterior; LAT:lateral; POST: posterior

Differences in LUS at T0 and T1 were not significant (*P* > 0.05). Lung ultrasound scores in the UD group were significantly lower than those in both the SI group and the C group at T2 (7.67 ± 1.15 versus 9.70 ± 102, difference, -2.03 [95% confidence interval, -2.77 to -1.29], *P* < 0.001; 7.67 ± 1.15 versus 11.73 ± 1.96, difference, -4.07 [95% confidence interval, -4.81 to -3.33], *P* < 0.001;, respectively). The intergroup differences were sustained until 30 min after tracheal extubation (9.33 ± 0.96 versus 11.13 ± 0.97, difference, -1.80 [95% confidence interval, -2.42 to -1.18], *P* < 0.001; 9.33 ± 0.96 versus 10.77 ± 1.57, difference, -1.43 [95% confidence interval, -2.05 to -0.82], *P* < 0.001;, respectively). The SI group had a significantly lower LUS than the C group at T2 (9.70 ± 1.02 versus 11.73 ± 1.96, difference, -2.03 [95% confidence interval, -2.77 to -1.29], *P* < 0.001), but the benefit did not persist at T3. (Table 2). Lung ultrasonic images of one representative patient in each group in the lateral region at different time points are displayed in Fig. 3. There was a significant difference in the LUS of the lateral zone between groups, whereas the LUS of the anterior and posterior zones showed no differences. For clarity, the temporal evolution of the mean LUS per quadrant per group was analysed (Fig. 4). Figure 5 shows a visual model of the temporal evolution of the mean LUS results per quadrant per group. Compared with the UD group, both the C group and the SI group displayed more dark-colored compartments (the darkness or brightness of the color corresponds to the LUS) during the perioperative period.

**Table 2:** Temporal evolutions of the lung ultrasound score

Time Point	C group	SI group	UD group
T0	1.27 ± 0.64	1.30 ± 0.70	1.30 ± 0.60
T1	4.00 ± 0.83	3.83 ± 0.83	3.63 ± 0.85
T2	11.73 ± 1.96	9.70 ± 1.02^a^	7.67 ± 1.15^*#^
T3	10.77 ± 1.57	11.13 ± 0.97	9.33 ± 0.96^*#^

^* ^Denotes significant difference compared with C group (*P* < 0.05)

^# ^Denotes significant difference compared with SI group (*P* < 0.05)

### Secondary outcomes

The differences in gas exchange, ventilator parameters, and hemodynamics among groups were not statistically significant at T1 (*P* > 0.05). At T2, the UD group had lower driving pressure, airway peak pressure, and airway platform pressure than the SI group and the C group. Furthermore, the oxygenation of both the UD group and the SI group was better than that of the C group at the T2 and T3 time points (*P* < 0.05, Table 3).

**Table 3:** Ventilator parameters, Gas exchange and Hemodynamics

	T1			T2			T3		
	C group	SI group	UD group	C group	SI group	UD group	C group	SI group	UD group
VT	447.50 ± 54.25	433.03 ± 49.69	421.08 ± 55.80	432.20 ± 52.32	418.23 ± 46.90	409.90 ± 54.52			
VT(PBW)	8.80 ± 0.76	8.62 ± 0.66	8.44 ± 0.95	8.49 ± 0.77	8.33 ± 0.64	8.20 ± 0.92			
PEAK	15.87 ± 1.31	15.67 ± 1.27	15.93 ± 1.23	27.40 ± 2.44	24.23 ± 2.25^a^	23.30 ± 1.56^a#^			
Plat	14.30 ± 1.18	14.23 ± 1.25	14.20 ± 1.13	24.73 ± 2.21	22.00 ± 1.74^a^	20.33 ± 1.49^a#^			
Driving pressure	9.77 ± 1.96	9.77 ± 1.61	9.60 ± 1.45	24.73 ± 2.21	17.00 ± 1.74^a^	15.33 ± 1.49^a#^			
SaO2	96.83 ± 1.42	97.13 ± 1.63	96.93 ± 1.51	100.00 ± 0.00	100.00 ± 0.00	100.00 ± 0.00	94.90 ± 2.16	97.57 ± 1.76^a^	96.67 ± 1.30^a^
PaCO2	36.93 ± 2.57	36.80 ± 2.85	35.60 ± 2.69	41.30 ± 2.68	40.90 ± 2.11	40.67 ± 2.48	39.63 ± 3.02	40.00 ± 1.66	39.73 ± 2.27
PaO2	492.13 ± 22.06	497.23 ± 36.25	501.33 ± 32.80	261.70 ± 25.72	283.97 ± 33.85^a^	300.33 ± 37.07^a^	81.80 ± 32.67	92.80 ± 9.13^a^	96.87 ± 11.21^a^
Oxygenation Index	492.13 ± 22.06	497.23 ± 36.25	501.33 ± 32.80	435.80 ± 42.81	475.90 ± 57.46^a^	500.23 ± 61.79^a^	344.83 ± 37.27	430.30 ± 42.45^a^	456.87 ± 56.79^a^
Mean artery pressure	76.60 ± 11.38	81.07 ± 11.24	76.73 ± 9.69	76.73 ± 9.92	82.17 ± 10.37	77.87 ± 13.61	84.77 ± 9.57	85.83 ± 9.19	83.77 ± 8.92
Heart rate	71.87 ± 10.27	72.70 ± 11.42	72.90 ± 11.85	70.87 ± 9.95	77.00 ± 10.33	74.67 ± 10.35	78.10 ± 9.17	80.20 ± 7.36	78.27 ± 9.4

^a^ Denotes significant difference compared with C group

^#^ Denotes significant difference compared with SI group (*P* < 0.05)

At T1 and T2, the number of lung recruitment manoeuvres among all groups was statistically significant (χ2 = 45.882, *P* = 0.00). The number in the SI group was 1, while the number in the UD group was mostly 2. At the T1 time point, no significant difference was observed in maximal inflation pressure during lung recruitment manoeuvres among the groups (χ2 = 3.125, *P* = 0.077, Table 4).

**Table 4:** Number of recruitment manoeuvres

	T1		χ2 *P* value		**T2**		χ2 *P* value	
	SI group	UD group			SI group	UD group		
Number of RMs								
0	0(0.00%)	0(0.00%)	45.88	0.00	0(0.00%)	0(0.00%)	49.09	0.00
1	30(100.00%)	4(13.33%)			30(100.00%)	3(10.00%)		
2	0(0.00%)	14(46.67%)			0(0.00%)	13(43.33%)		
3	0(0.00%)	8(26.67%)			0(0.00%)	7(23.3%3)		
4	0(0.00%)	4(13.33%)			0(0.00%)	6(20.00%)		
5	0(0.00%)	0(0.00%)			0(0.00%)	1(3.33%)		
Maximal inflation pressure(cmH_2_O)								
30	30(100.00%)	18(90.00%)	3.13	0.08	30(100.00%)	13(65.00)	12.21	0.00
35	0(0.00%)	2(10.00%)			0(0.00%)	5(25.00%)		
40	0(0.00%)	0(0.00%)			0(0.00%)	2(10.00%)		

There were 2 cases of postextubation hypoxemia in the C group, and deoxygenation saturation was between 90 and 95% in most patients, while peripheral oxygen saturation was higher than 95% in both the SI group and the UD group, indicating that oxygenation was improved after extubation after ventilation intervention (2 = 36.47, *P* < 0.001, Table 5).

**Table 5:** Peripheral oxygen saturation after extubation

	C group (*n* = 30)	SI group (*n* = 30)	UD group (*n* = 30)	**χ2**	*P* Value
SpO_2_ ≤ 90%	2 (6.70%)	0 (0.00%)	0 (0.00%)	36.47	0.00
90% < SpO_2_ < 95%	21 (70.00%)	3 (10.00%)	5 (16.70%)		
SpO_2_ ≥ 95%	7 (23.300%)	27 (90.00%)	25 (83.30%)		

No between-group differences were observed in pneumonia, pleural effusion, atelectasis, extubation failure, postoperative pain scores, or length of hospital stay after surgery within the 48 h postoperative period (Table 6). No other respiratory complications occurred during the study period.

**Table 6:** Postoperative complications and hospital stay

	C group (*n* = 30)	SI group (*n* = 30)	UD group (*n* = 30)	χ^2^ /F	*P* value
Postoperative pneumonia ^a^	1(3.33%)	1(3.33%)	1(3.33%)	0.00	1.00
Postoperative pleural effusion ^b^	2(6.67%)	2(6.67%)	1(3.33%)	0.42	0.81
Atelectasis in PACU ^c^	3(10.00%)	2(6.67%)	2(6.67%)	0.40	0.86
Postoperative atelectasis ^c^	3(10.00%)	4(13.33%)	3(10.00%)	0.23	0.89
Extubation failure	0(0.00%)	0(0.00%)	0(0.00%)	-	-
Postoperative pain scores	3.90 ± 1.16	3.93 ± 0.94	3.77 ± 1.17	0.20	0.82
Length of hospital stay after surgery(days)	5.13 ± 1.279	5.33 ± 1.028	5.53 ± 1.252	0.85	0.43

Data are presented as mean ± SD or number (%)

^a^ Pneumonia defined as cough with body temperature at least 37.5℃ or WBC > 10 × 109/L, also is confirmed by chest radiograph

^b^ Pleural effusion is confirmed by chest radiograph

^c^ Atelectasis is also confirmed by chest radiograph

## Discussion

In previous studies, the recruitment manoeuvre, which is one component of the lung-protective ventilation strategy, has been proven to be effective in reducing perioperative atelectasis and improving oxygenation among infants, children and adults during laparoscopic surgery [8, 9, 11, 12]. There are many methods for recruitment manoeuvres, including a sigh, pressure control method, sustained inflation and so on. What is the best method of RMs? The answer to this question is still uncertain. Zhao et al [23] and Karsten et al [24] demonstrated that high RMs could lead to (re)opening collapsed lung units and an increase in ventilation-independent areas. Eronia et al [25] showed that sustained inflation RMs lead to improved oxygenation and reduced driving pressure. De Matos et al [26] demonstrated that pressure control RMs could efficiently reverse hypoxemia and most collapsed lung units in patients with acute respiratory distress syndrome. In recent research, ultrasound-guided RMs for atelectasis have been validated and gradually applied to lung-protective ventilation strategies [27, 28]. LUS had reliable performance in postoperative atelectasis, with a sensitivity of 87.7%, specificity of 92.1%, and diagnostic accuracy of 90.8% [29]. Studies by other scholars also showed that a higher LUS score indicated more severe atelectasis [30]. LUS provides a fast, reliable and radiation-free method to identify peri-operative atelectasis in adults. Therefore, we tested the effects of ultrasound-guided RMs compared with sustained inflation RMs on atelectasis in our trial. Our data showed that the UD group had a lower LUS and a higher PaO_2_/FiO_2_ratio than the SI group and the C group at T2 in adult patients undergoing laparoscopic gynecological surgery (< 2 h) who were anesthetized, and the UD group also had reduced atelectasis during the perioperative period. The benefit was sustained until 30 min after tracheal extubation in the UD group, which is in line with previous trials [12].

Otherwise, the SI group did not result in a lower LUS than the C group at T3 in this study. This result may account for the different frequencies of RMs; more than 90% of patients in the UD group had more than two RMs, while only one RMs was observed in the SI group. Almarakbi et al [31] measured the effects of different frequencies of RMs and noted that the benefit of intraoperative PaO_2_, pulmonary compliance, and postoperative PaO_2_was temporary in the single alveolar RM but persisted in the repeated-measures alveolar RM. As the extent of alveoli collapse and the responses to recruitment manoeuvres vary among individuals, it has been suggested that individualized assessment is needed [32]. The responses to recruitment manoeuvres were dynamically monitored by ultrasound in real time, and the frequencies of RMs and driving pressure were more individualized in the UD group. The ultrasound-guided alveolar recruitment manoeuvre was conducted under an effective visual angle, which benefited the patients more during the perioperative period.

Additionally, we analysed aeration loss in different quadrants during general anesthesia in our trial. Our data showed that lung aeration in the infereoposterior and inferolateral regions still worsened after the recovery of automatic respiration. Our results demonstrated that the between-group differences in LUS were sustained until 30 min after tracheal extubation, whereas the incidence of atelectasis confirmed by chest radiography in both the PACU and the 48 h postoperative period was not different among groups. We also noticed that atelectasis occurred more frequently in the gravity-dependent lung zones, which agreed with the findings of previous studies [33, 34].

Similar to previous trials [28, 35], both the UD group and SI group (the use of RM) led to a significant increase in pulmonary compliance and lowered driving pressure compared with the C group (no RM use) in our trial. A meta-analysis suggested that PPCs were more correlated with driving pressure than any other ventilatory parameter, which may change ventilation strategies [36]. In addition, changes in individual parameters, such as tidal volume, Pplateau or PEEP, were not significantly correlated with survival [35, 37].

In the three groups, no intergroup differences were observed in postoperative complications, postoperative pain, or length of hospital stay after surgery. This may be explained by normal preoperative pulmonary function and short operation time in all patients. While reducing perioperative atelectasis alone may provide lung protection, direct causality is unproven, [38, 39] and this remains controversial [40, 41].

Our study has several strengths. First, although CT was considered to be the “gold standard” for evaluating lung reaeration, determining the degree of reaeration is still challenging. In the present study, we demonstrated that ultrasound-guided RMs could visually (re)open collapsed lung parts and that the degree of atelectasis can be effectively evaluated by using a validated semiquantitative score [21]. Second, perioperative lung aeration changes in the three groups were tracked in this study. Our data showed that the UD group not only safely and effectively evaluated perioperative atelectasis but also had a better effect on (re)opening collapsed lung units than the other groups. It provides insight into lung-protective ventilation strategies commonly used by anaesthesiologists [42] Third, all ultrasound images were evaluated offline by a seasoned lung sonographer who was blinded to allocation details to reduce errors.

There are also some limitations in our trial. First, our study is a small sample and single-center study, and the results still need to be verified by a multicenter and large sample study. Second, LUS cannot be used for assessing lung overdistention while carrying out lung recruitment manoeuvres. Although we did not observe any adverse events in this trial, we still need to closely monitor the patient's vital signs during the implementation of RMs. Third, we included healthy female patients, although sex was not an independent factor related to the development of atelectasis [43]. Thus, our conclusions are limited to female patients with normal pulmonary function, and further studies should be performed on patients with medium- and high-risk factors.

In conclusion, ultrasound-guided RMs can reduce perioperative aeration loss and improve oxygenation during general anesthesia, and some degrees of aeration loss were sustained 30 min after tracheal extubation. Furthermore, these effects of ultrasound-guided RMs on atelectasis are superior to those of sustained inflation RMs.

## Data Availability

The datasets generated during and/or analysed during the current study are available from the corresponding author on reasonable request. All data generated or analysed during this study are included in this published article.

## Abbreviations

RMs: Recruitment manoeuvres
PPCs: Postoperative pulmonary complications
LUS: Lung ultrasound score
CI: Confidence interval
PEEP: Positive end-expiratory pressure
ASA: American Society of Anesthesiologists
PACU: Postanesthesia care unit
MAP: Mean arterial pressure
Ppeak: Peak airway pressure
FIO_2_: Fraction of inspired oxygen
SpO_2_: Peripheral oxygen saturation
PBW: Predicted body weight
ANOVA: Analysis of variance
PaO_2_: Arterial partial pressure of oxygen
